# Inclusive assessment in health professions education: Balancing global goals and local contexts

**DOI:** 10.1111/medu.15535

**Published:** 2024-09-10

**Authors:** Gabrielle M. Finn, Joanna Tai, Vishna Devi Nadarajah

**Affiliations:** ^1^ School of Medical Sciences, Faculty of Biology, Medicine, and Health University of Manchester Manchester UK; ^2^ Centre for Research in Assessment and Digital Learning (CRADLE) Deakin University Melbourne Australia; ^3^ Newcastle University Medicine Malaysia, Faculty of Medical Sciences Newcastle University Iskandar Puteri Malaysia

## Abstract

**Context:**

In this article, we draw upon diverse and contextually different experiences of working on inclusive assessment, with the aim of bridging and enhancing practices of inclusive assessments for health professions education (HPE) within universities. Instead of juxtaposing our views from three countries, we combine our perspectives to advocate for inclusive assessment.

**Discussion:**

Creating an inclusive assessment culture is important for equitable education, even if priorities for inclusion might differ between contexts. We recognise challenges in the enactment of inclusive assessment, namely, the notion of lowering standards, harming reliability and robustness of assessment design and inclusion as a poorly defined and catchall term. Importantly, the lack of awareness that inclusion means recognising intersectionality is a barrier for well‐designed inclusive assessments. This is why we offer considerations for HPE practitioners that can guide towards a unified direction of travel for inclusive assessments. This article highlights the importance of contextual prioritisation and initiatives to be considered at the global level to national, institutional, programme and the individual level. Utilising experience and literature from undergraduate, higher education contexts, we offer considerations with applicability across the assessment continuum.

**Context:**

In this state of science paper, we were set the challenge of providing cross‐cultural viewpoints on inclusive assessment. In this discursive article, we focus on inclusive assessment within undergraduate health professions education whilst looking to the wider higher education literature, since institutional policies and procedures frequently drive assessment decisions and influence the environment in which they occur. We explore our experiences of working in inclusive assessment, with the aim of bridging and enhancing practices of inclusive assessments for HPE. Unlike other articles that juxtapose views, we all come from the perspective of supporting inclusive assessment.

We begin with a discussion on what inclusive assessment is and then describe our contexts as a basis for understanding differences and broadening conversations. We work in the United Kingdom, Australia and Malaysia, having undertaken research, facilitated workshops and seminars on inclusive assessment nationally and internationally. We recognise our perspectives will differ as a consequence of our global context, institutional culture, individual characteristics and educational experiences. (*Note that individual characteristics are also known as protected characteristics in some countries*).

Then, we outline challenges and opportunities associated with inclusive assessment, drawing on evidence within our contexts, acknowledging that our understanding of inclusive assessment research is limited to publications in English and currently tilted to publications from the Global North. In the final section, we then offer recommendations for championing inclusion, focussing firstly on assessment designs, and then broader considerations to organise collective action.

Our article is unapologetically practical; the deliberate divergence from a theoretical piece is with the intent that anyone who reads this paper might enact even one small change progressing towards more inclusive assessment practices within their context.

## Abstract 2

In this article Finn, Tai, and Nadarajah explore diverse and contextually different experiences of working on inclusive assessment, with the aim of bridging and enhancing practices within HPE.

## 1 INTRODUCTION

It is the experience of the authors that one fundamental issue with assessment design is the assumption that assessment is a neutral act. Assessment is neither culture‐free nor value‐neutral; its associated procedures, structures and systems codify the norms and values of cultural, disciplinary and individual knowledge hierarchies.^1^ All this goes to say that assessment is many things to many people, including a relation of power.^2^ The design and implementation of assessments is controlled by those in positions of authority including stakeholders such as educators, employers, patients and policymakers. Yet, rarely do students have a voice in assessment decisions. Assessors become gatekeepers, and this enables their position of power. Gatekeeping in assessment can be viewed positively, through the lens of quality assurance or negatively—as those who are in power call the shots and keep ‘others’ out. Thus, when we turn our minds to inclusive assessment, we must be cognisant of culture, gatekeeping and power in assessment.

For those involved in undergraduate health professions education (HPE), whether students, practitioners or leaders, high‐quality assessments are a priority. Inclusion often may not be at the top of the priority list, and, as such, historically assessment has struggled to meet the needs of student diversity, especially from the viewpoint of disabilities.^3^ There is also variability in what *inclusion* means, and looks like, across the aforementioned stakeholders in assessment. Given the multiple stakeholders and rapid changes seen in the philosophy, practice and delivery of assessments, we as leaders and practitioners need to become better at assessing our current practice of assessments, deliberating inclusive assessments and recognising the risks and significance of any changes.

## 2 WHAT IS INCLUSIVE ASSESSMENT?

‘Originally focusing on disabled students, inclusive assessment aims to tackle assessment at point of design – looking at all aspects, from the development of marking criteria to method and mode of feedback – to ensure the ways in which we assess do not exclude students’.^1^ Inclusive assessment is an issue for the health professions because of its potential for multiple interpretations and ways of implementation in practice. University HPE is the entry point for diverse practitioners and thus is a logical place to begin this conversation. At its heart, inclusive assessment is concerned with equity in assessment—but equity can be thought of along many different dimensions. Inclusion was originally about involving disabled students in ‘mainstream’ education, focussing on reasonable adjustments made during learning and assessments according to individual needs. Social inclusion—focussing on aspects of identity such as gender, sexuality and ethnicity—has also become part of the agenda in more recent times, broadening both the scope and expectations of what should be done for assessment to be inclusive.^4^ Recent work suggests the focus should be on the design of assessment, rather than particular characteristics of the student, with the aim to not exclude or unfairly disadvantage some students. Inclusive assessment design offers all students equal opportunities to demonstrate their learning and achievements, and it reduces the need for individual adjustments to assessments.^5^ Considerations pertaining to the inclusivity of an assessment might include consideration of the conditions that the assessment task will be undertaken within, developing marking criteria, the writing of instructions and the writing of the assessment item itself.^6^ Further, inclusive assessment focuses on the need for continuous assessment and evaluation achieved through formative and summative assessments.^4^ As assessment practitioners, designers and researchers, our experiences suggest that inclusive assessments if not addressed comprehensively in HPE can be a potential risk and significantly impact assessment outcomes. These risks include the lack of defensible policies or guidelines to practice inclusive assessments, increased workload for educators and students and unfairly preventing learners from progressing and reinforcement of systemic bias that disadvantages individuals and their wellbeing.

Whilst inclusion in assessment might feel like a fringe issue to some, equity within assessment could be considered as central wicked problem. Wicked problems are defined by their inherent uncertainty, conflict, tensions and contextual influences.^7^ Though in many contexts inclusion through participation might be the current focus, we argue inclusion in assessment—and its functions in admissions, progression and certification—are core to ensuring HPE is successful.

Inclusive assessment does not feature as prominently in the HPE literature as it does within the wider higher education context. Empirical evidence, even within the wider context is lacking. For example, a review by Tai et al. reported only one study examining the effects of inclusive assessment on student learning.^8^ Other examples include advice on supporting disabled learners through digitised assessments,^9^ qualitative explorations of perceptions of causes of differential attainment^10^, ^11^ and theoretical articles on equity within assessment.^7^ The paucity of empirical studies on inclusive assessment is likely as a consequence of HPE assessment being so high‐stakes. Literature within the North America context speaks to equitable or fair assessment,^7^, ^12^ seemingly used synonymously within inclusive assessment.

## 3 CONTEXTS FOR INCLUSION IN HPE

The United Kingdom, like many countries, has a strong commitment to equality, diversity and inclusion (EDI), including widening access to universities for groups from underrepresented populations.^13^ These populations include students leaving social care provision, students from particular ethnic backgrounds, those from lower socio‐economic status households or those who are first in their family to enter higher education.^13^ Historically, admitting students from more diverse backgrounds into higher education was the primary goal, with limited consideration being given to enabling students to thrive once admitted, or progress into gainful employment. This is evidenced by the perpetuation of awarding gaps and differential attainment,^14^, ^15^ observed across the continuum of HPEs and careers.^15^ The expectations from the Office for Students in the United Kingdom are clear, ‘to ensure that all students, from all backgrounds, with the ability and desire to undertake higher education, are supported to access, succeed in, and progress from higher education’.^16^ Tackling inequalities in HPE and training is important to ensure patients can benefit from a diverse workforce.^15^, ^17^

Australia is also increasingly recognising the importance of inclusive education, with the Australian Centre for Student Equity and Success established in 2013 to support efforts within higher education. Following ‘A fair chance for all’, a national framework for equity in higher education published by the federal government in 1990, there has been compulsory collection of data about nominated groups: people from low socio‐economic backgrounds, Indigenous Australians, people from regional and remote areas, people with disabilities, people from non‐English‐speaking backgrounds and women in nontraditional areas. This affords analysis and monitoring at a national level. Whilst there has been some success in widening access and participation, the success of equity groups has been mixed.^18^ Legally, the federal Disability Discrimination Act^19^ and Disability Standards for Education^20^ require institutions to enable access for those with disabilities. However, other equity groups and (in the UK terminology) protected characteristics fall under state‐based legislation, which covers education contexts, so whilst there are overlaps, there can also be gaps in coverage.^21^ In the context of health care, supporting Indigenous health and reducing geographic disadvantage have been strong drivers for encouraging equity group students from those communities to become health professionals.

Malaysia's commitment to the inclusive education movement is explicit in the ‘Malaysia Education Blueprint 2013‐2025’.^22^ Inclusive education started in the 1990s with focus on special needs learners in both school and higher education settings, moving to a broader agenda of accessible education for all learners through adoption of its new Inclusive Open Educational Resources policy in 2022. Widening access in medical education has also been a priority. Policies on medical school entry and available student loans or scholarships are centrally governed with quotas for students, based on geographical location, race (including those from indigenous groups) and socio‐economic background.^23^ Given the diversity of students entering medical education in Malaysia, programme accreditation emphasises appropriate developmental or remedial support to assist students, importance of student representation in curriculum management and availability of personal and wellbeing support including access to religious and spiritual support services. Accreditation standards require programmes to show evidence of fair and transparent assessment regulations and practices. This requirement has become more important in the recent years with increasing numbers of new medical graduates opting out of practicing medicine.^24^

All contexts highlight that graduating health care practitioners without attending to equity is simply not enough, and that what is foregrounded to be important for inclusion differ depending on local history and priorities. Assessment ‘has been shown to be the single most important component that influences student learning and education in general’,^25^ and so, from a learning perspective, creating an inclusive assessment culture is arguably the most important way to ensure equity and inclusion for students and their success: Inclusive assessment matters.

Inclusive assessment also matters from an assessment of learning or certification perspective, to ensure safe practice. As a field, we have led the way in terms of psychometrics, and the scrutiny under which we place assessments.^26^ For example, the utility equation coined by van der Vleuten^19^ calls for an evidence‐based approach when assessing the reliability and validity of assessments. HPE also leads in terms of rigour in contemporary methods of standard setting.^27^ We suggest that inclusion should be a central consideration in assessment validity. If an assessment cannot distinguish between students who are capable, and those who are not, because of irrelevant characteristics, then how can we make claims of validity?

## 4 REFLECTIONS ON THE CHALLENGES AND OPPORTUNITIES IN INCLUSIVE ASSESSMENTS

There are a number of forces that come into play when considering inclusive assessment. We have identified the following challenges and opportunities in inclusive assessments based on our experiences of conducting workshops with faculties at our own institutions, regional and international conferences. The challenges highlighted here were commonly cited by participants in spite of the various geographical settings of our workshops.

### 4.1 ‘Lowering standards’

The biggest objection asserted with the notion of implementing inclusive assessment is that attempts to be inclusive will ‘dumb down’ assessments, making them ‘easier’ to pass. This concern arises from a deficit perspective of equity group students^28^: Instead, we suggest that, like for all students, the focus should on assessing what students are actually capable of, acknowledging the many possible pathways an individual might take to arrive at an appropriate and safe course of action. Challenges arise in the context of HPE where there is often not one ‘correct’ answer. Inclusive assessment does not equate to lowering academic standards and is argued to be a valuable tool in addressing awarding gaps.^29^, ^30^ Adopting principles of inclusive assessment design can, in fact, maintain standards whilst also generating more opportunities for students to demonstrate the breadth and depth of their learning.^30^ Evidence specifically supports the need for authentic assessment, as one tool in the inclusivity arsenal. Sotiriadou et al. reported that the more relevant an assessment task was to real‐world scenarios, the less likely students are to engage in misconduct.^29^ In the United Kingdom, experience tells us that any solution that supports academic integrity is popular with faculty, demonstrated by the 72% growth in EdTech in the United Kingdom in 2020.^31^ In Malaysia, experience suggests that solutions that are explicit in the health programme standards and driven by regulatory requirements are implemented by faculty. Diverse students also recognise the need for standards and seek to demonstrate their capabilities, rather than wanting any form of special treatment.^32^

### 4.2 ‘Harming reliability and robustness of assessment design’

Alongside concerns about standards, we have encountered claims that designing for inclusion risks the reliability and robustness of assessment. However, no single assessment task can effectively capture student knowledge,^33^ and a shift to programmatic assessment is likely to improve the reliability of decisions made in assessment.^34^ In HPE, there is a cultural overreliance on a limited number of assessments types, which restricts the opportunities for students to demonstrate the full extent of their learning across knowledge, skills and attitudes. Faculty are also often socialised into assessing in the way in which they themselves were assessed,^35^ rather than assessment design being a rational process There may also be inherent bias when assessments are designed, since they are often designed by those who succeeded in their education, those who had privilege, unlikely to be those from marginalised groups. This is often referred to as ‘survivor bias’ in other fields, where only those who survived a process are considered, not those who failed. For example, teams planning Objective Structured Clinical Examinations (OSCEs) are typically constructed of clinicians who have qualified by passing their own OSCE assessments, thus implicit biases may perpetuate. Within UK culture, the written essay remains the most dominant form of assessment.^36^ In order to truly design inclusive assessment, a greater variety of assessment formats need to be used—although this is easy to advocate for in a country such as the United Kingdom where the cost and resource implications are of less concern. In Malaysia, different assessment formats are available and applied for outcomes related to cognitive skills; the challenge is the resistance to implement and formally assess noncognitive skills, for example, ethics, empathy, collaboration and communication. Thinking more broadly about assessment design to encompass the preparation for the task and feedback post, the task can also offer possibilities for inclusion: Australian students have suggested that even revising the instructions for assessment processes for clarity, not presupposing facility with a particular assessment genre, could also represent steps towards inclusion.^37^ This may improve robustness and reliability, since students are more likely to have similar opportunities to engage in the learning related to the task.

### 4.3 ‘Inclusive means simply giving more options’

Inclusive assessment is sometimes conflated with choice, optionality or alternative assessment. Optionality in assessment refers to an assessment where students are able to make some sort of choice, for example, they could choose to submit a poster, an essay or a video.^38^ Where students have the option to choose between different assessment types, examples include different submission formats, team or individual approaches to assessment and the option to choose specific assessment types, or select from a range of topics. This offers students some agency within their assessment, such that they may choose the best way to demonstrate their capabilities, for example, reducing social anxiety relating to presenting to an audience or minimising the impact of dyslexia through not requiring a written format. However, mode of delivery is only a small part of the issue that makes some assessments inequitable. The literature reports a range of benefits to optionality in assessment including a positive impact on grades when students are given choice in assessment,^38^ attributed to students' increased motivation or enhanced wellbeing. Where studies did not find an improvement in grades, there were however improvements in other aspects of learning, for example, subject satisfaction.^39^ We have encountered questions regarding the validity of comparing ‘apples with pears with bananas’. The backdrop of regulated programmes makes assessment teams unwilling to consider choice in assessment, even if student outcomes are improved. Reflecting on practices in our settings, we find that optionality is usually used for low‐stakes student electives or student‐led projects, and the assessment outcomes are not used summatively, which presents it as a ‘poor cousin’ to other assessments. A programmatic approach may be useful to map required outcomes if considering incorporating choice in high‐stakes assessments: Where and how might students be asked to demonstrate required learning outcomes, and how might they be scaffolded through formative and low‐stakes tasks to achieve these outcomes? There may also be dilemmas relating to educator workload when additional topics or formats require additional time to assess. The United Kingdom is moving towards increasing optionality within assessment due to the student benefits^38^ but particularly in response to a recent High Court judgement where a student sadly took their own life due to the stress of an oral assessment.^40^, ^41^ A concern when introducing optionality in assessment can often be standard setting and the notion of comparability. Further, as HPE in many geographies moves towards national licensing examinations, such as the USMLE in the United States or the MLA in the United Kingdom, the appetite to for offering variation is diminishing. Such tests have already been critiqued for inherent bias,^42^ which could lead to greater caution and less willingness to have and deviation in approach.

### 4.4 ‘Inclusion as a catchall term’

A major challenge is that inclusion has become a catchall term, used equally with respect to disability inclusion, and social inclusion in higher education.^5^, ^43^ Whilst both forms are important, the priorities and ways to achieve inclusion, respectively, may differ. Disability inclusion encompasses many groups including physical disabilities, learning disabilities and mental and physical health conditions. Social inclusion encompasses students from low socio‐economic backgrounds, women in nontraditional areas, indigenous and First Nations peoples,^5^ as well as with respect to sexuality and gender.^44^ A further challenge is that those within these equity groups are not homogenous,^5^ just as any student cohort being assessed is not homogenous. This means that we must think about ensuring fairness and objectivity in inclusive assessment practice across many intersecting dimensions. It is our experience that the use of inclusivity as a catchall term means that we frequently make assumptions about *who* we are including, *why* it is important, and *how* we might achieve this. Calls for inclusion have also been drawn into loftier aspirations of assessment for social justice, which has been described as both a process and a goal.^45^ McArthur describes it as a two‐pronged term: (i) the justice of assessment practices and processes within higher education and (ii) the role of assessment in nurturing forms of learning that will promote greater social justice in society as a whole.^45^ This framing considers society as a whole; here, we argue that we need to start by considering how individuals—our students—should be supported.

Furthermore, calls for inclusion and the proactive design of assessment cannot entirely erase the need for longstanding accommodations (sometime referred to as contingent assessments or adjustments) for students with disabilities, such as increased time allowances, provision of an amanuensis, alternative formats or allowing the use of assistive technology. Institutions must continue to be responsive to individual students' needs, all the whilst identifying and implementing possibilities for inclusive assessment design.

## 5 DIFFERENT WORLDVIEWS ON INCLUSION AND THE CHALLENGE OF INTERSECTIONALITY

We began this article describing the authors' different contexts, and that we are all champions for inclusion, and inclusive assessment. There are of course some aspects where, despite our personal views, our cultures and contexts differ with respect to inclusion. It is important to acknowledge these given the global classrooms that dominate within HPE.

As Tai et al. describe, assessment for inclusion recognises the many spectra of diversity, and the intersections of these spectra need consideration in both what and how we assess. This intersectionality is where our experiences diverge{Tai, 2022 #7077}. When we speak of inclusion, as we have posited above, we speak of the structural, systemic and cultural bias and disparities associated with assessment on a range of axes. How these disparities are experienced will very much depend on the intersecting identities and protected characteristics of the students. For example, a LGBT, Black student will have a very different assessment experience to a neurodivergent White student, and both of these students would have a different experience again in a different country. Despite intersectional differences, assessment tools and structures rarely flex.

Within the United Kingdom, such characteristics are protected, as evidenced by the Equality Act 2010. This means that assessors also need to be mindful of the diverse and intersecting protected characteristics of those completing assessments but also those that feature within them. For example, students in the United Kingdm have raised concerns over about heteronormative assessment items or stereotyping on the basis of gender, age and ethnicity.^38^ In comparison, Malaysian society is more accepting towards heteronormative relationships, and homosexual partnerships cannot be legalised; hence, health professions educators are cautious not to reinforce negative stereotypes based on age, gender identity, ethnicity and sexual orientation in both the learning and assessment environment. Nevertheless, writing assessment items that may be deemed inclusive in the western perspective can be problematic. For example, students for all official purposes are asked to identify their gender as male or female; there are no options for doing otherwise. Further, literature form the Middle East suggest that translating policy into practice poses significant challenges, with deeply entrenched perspectives influencing public perceptions of individuals with disabilities in Islamic countries.^46^ The basis of these issues is centred upon the medicalisation of disability within Islamic countries, as opposed to the social model adopted in western culture.^46^ We cannot presume to know what inclusion should look like in every context around the world. We emphasise instead that whilst the general principle of inclusive assessment should stand, we should all be striving towards inclusion in assessment, which is nuanced to the local contexts and considerations at play.

Inclusion for students is not only about their ability to participation in assessment but also to see themselves represented within assessment content. This is where our experiences differ significantly. All three contexts, the United Kingdom, Australia and Malaysia are working to address representation, although the progress may vary.^47^ In Malaysia, students with disabilities or neurodiversity are not selected out of higher education or HPE, but because the awareness and resources to support is lower (as compared to highly developed countries) from primary to tertiary education, representation or evidence of representation especially in HPE is lacking.^48^ It is important to consider representation within inclusion; without doing so, we risk further perpetuating biases and oppression.^49^ One way to address this is to consciously cocreate assessment content with students.^50^

## 6 INCLUSIVE ASSESSMENT STARTS AT THE POINT OF DESIGN

Inclusive assessment is not simply about making individual adjustments for each student's unique needs during the assessment process: This would be exhausting. Instead, it is about taking a proactive stance right from the point of design. Such assessment design ensures that every student has the same chance to show what they have learned and achieved, thereby decreasing the necessity for tailored adjustments to assessments subsequently.^5^ As described earlier, there are implicit cultural assumptions and biases with assessment and its processes.^4^ We accept, because of this, there is a risk of unconscious bias. Many inclusivity issues can be accounted for if inclusion is considered at the point of design. In Table 1, we offer four problem areas relating to inclusive assessment and give examples of proactive actions towards inclusion, which take place at the design level, rather than making individual adjustments.

**TABLE 1:** Examples of assessment issues experienced across the United Kingdom, Australia and Malaysia.

Assessment problem area	Example of dilemma	Course of action
Inclusive language within assessment content	Clinical vignettes referred solely to women menstruating or women giving birth, when people who do not identify as women may do these things.	A UK university has introduced inclusivity reporting for students. They can log concerns over assessment items that are not inclusive. There is a workflow within the institution to investigate and resolve issues flagged.^51^ For the two examples provided, language was changed accordingly.
	Multiple choice assessment items use language that denigrate non‐heterosexual relationships, for example, the male patient *admitted to* having sex with a male partner	
Timing of assessments	Assessment timings do not consider cultural or religious holidays and festivals. Students who have work or caring responsibilities do not have the same freedom with regard to assessment deadlines. Midnight deadlines also favour students who are physically capable of staying up late	The Ministry of Higher Education Malaysia mandates that universities must not have assessments on the eve, day and 1 day post major religious festivals for students to have options to celebrate with their families. At an Australian university, the official timing for assignment submission is 8 PM, but no penalty is taken between 8 PM and midnight.
Assessment formats	High‐stakes summative assessment emphasises (or gives higher weight) to knowledge‐based written assessments. Assessment of groupwork is increasing commonplace with undergraduate programmes especially for community or research projects. Student groups are diverse where culture, nationality, gender dynamics, egos and work ethics can be significant barriers in promoting and evaluating groupwork	Incorporation of authentic elements, in which students need to apply their learning to ‘real‐world’ or simulated context Explicit support for students to familiarise them with types of assessment and how to go about them. For example, at an Australian university, explicit information in multiple formats is offered orienting students to the purpose, format and expectations of the task, including videos and where groupwork and peer assessment is required, ‘how to’ guides for peer feedback. In Malaysia all international undergraduate students are expected to attend short courses related to learning the local language and culture within the first 2 years of medical education, formative assessment for these courses are usually groupwork related community engagement and are useful to orientating students to the local community.
Equivalence of assessment judgements across contexts and assessors	In workplace‐based assessments across distributed teaching sites, students can feel that their learning and assessment opportunities are different and sometimes disadvantaged from other students. Assessor–student interactions are common in health professions education, since there is a requirement to observe, engage and make judgements based on interactions. Due to these personal interactions, assessment outcomes are sometimes challenged.	These are matters of assessment validity, so usual measures can help to address equivalence, Including diverse assessors, offering assessor training and feedback across sites and standardised assessment tools (e.g. rubrics). Training should generate consensus on standards, recognise diversity in ways to demonstrate those standards and be aligned to guidelines or code of conduct for examiners and made available to both examiners and students. Rotations across sites will also enrich the student experience. Students should have access links in which they can safely raise and report critical incidences.

## 7 CONTEXTUAL CONSIDERATIONS FOR PROMOTING INCLUSIVE ASSESSMENTS

**FIGURE 1:**
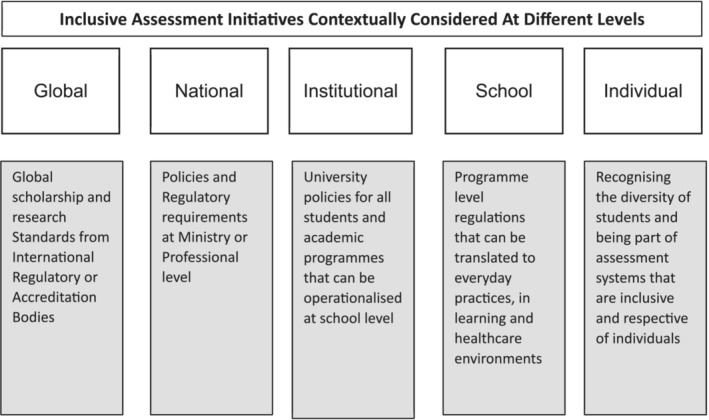
Inclusive assessment initiatives contextually considered at different levels.

Whilst we have offered some practical actions to in response to specific assessment problems, we also acknowledge that these may not be the priorities in all readers' contexts. This is because medical schools' populations are increasingly diverse and international with student exchange programmes and branch campuses. This may add additional challenges with straddling potentially misaligned sociocultural or regulatory factors in design. Therefore, Figure 1 also briefly outlines more general initiatives aimed towards inclusive assessment, illustrating how they might work together across different levels. These considerations highlight the importance of contextual prioritisation from the global level to the individual level. Recognising the limitations of practicing a ‘one size fits all’ solution at the global level, priorities should be widening global scholarship and research on Inclusive Assessments and developing general standards for practice. At the national level, both the government through respective ministries or professional bodies can develop policies or set regulatory requirements for inclusive practice at higher education or health care institutions. These national policies and regulatory requirements are developed into institutional policies that can be operationalised at school or programme level. At the same time institutions can drive initiatives across schools to create safe spaces for conversations and normalise inclusive assessments. At the school or programme level, care should be taken to understand the diversity of the student population in the health care learning environment, and proactively address student needs in alignment with professional practice and standards for HPE. Finally, at the individual level, inclusive interactions can make a significant difference to students' feelings of inclusion and belonging.^32^

## 8 CONCLUSIONS

Some steps towards inclusive assessment have already been made. However, the journey is far from complete: issues of bias, resource allocation and the balance between standardisation and individualisation persist. Assessments must adapt to local contexts whilst striving to meet international standards of education and equity, noting that nuances of societal views may not resonate globally. There is also increasing recognition that diverse assessor perspectives are beneficial. In the shift towards constructivist perspectives, we should be careful to consider if entrenched privileges and biases might also be perpetuated in the shift towards new systems of assessment. Failing to address such biases poses the risk of exacerbating awarding gaps and differential attainment.^10^, ^14^, ^15^

The cross‐pollination of ideas across the globe can develop more robust, adaptable and truly inclusive assessment practices that are sensitive to the needs of diverse student populations. Opening and continuing discussions at all levels from the global to the individual will be important to achieve this endeavour. The paucity of evidence as to the effectiveness of inclusive assessment within HPE needs addressing. The HPE community reliance on standardisation risks alienating students and further perpetuating inherent biases that disadvantage marginalised groups.

In moving forward, it is essential to embrace a collaborative global dialogue that respects the unique contributions and circumstances of each context. Such an exchange can inspire policies and practices that not only accommodate differences but also celebrate them as vital in the climate of global education. Ultimately, the goal is not to diminish the importance of local contexts but to weave them into a larger narrative that advocates for the success and recognition of every learner, regardless of their origin and personal characteristics. Without supporting diverse learners, the unique benefits of a diverse health care workforce will cease to exist. We advocate for addressing the lack of inclusion within assessment at the point of design, through cocreation with students.

## AUTHOR CONTRIBUTIONS

**Gabrielle M. Finn:** Conceptualization; investigation; funding acquisition; writing – original draft. **Joanna Tai:** Conceptualization; methodology; software; data curation. **Vishna Devi Nadarajah:** Conceptualization; methodology; validation; investigation; formal analysis; supervision; data curation; software.

## ETHICS STATEMENT

Not applicable.

## Data Availability

Data sharing not applicable to this article as no datasets were generated or analysed during the current study.

## References

[1] Hanesworth, P. Inclusive assessment; where next? 2019; Available from: https://www.advance-he.ac.uk/news-and-views/inclusive-assessment-where-next

[2] Burke PJ . Inclusive assessment: Recognising difference through communities of praxis. In: Assessment for Inclusion in Higher Education. Routledge; 2022:87‐97. doi:10.4324/9781003293101-11

[3] Nieminen JH . Assessment for inclusion: rethinking inclusive assessment in higher education. Teach High Educ. 2024;29(4):841‐859. doi:10.1080/13562517.2021.2021395

[4] Haneworth, P. Inclusive Assessment: Where Next? 2019; Available from: https://www.advance-he.ac.uk/news-and-views/inclusive-assessment-where-next

[5] Tai J , Ajjawi R , Bearman M , Boud D , Dawson P , Jorre de St Jorre T . Assessment for inclusion: rethinking contemporary strategies in assessment design. High Educ Res Dev. 2022;42(2):483‐497. doi:10.1080/07294360.2022.2057451

[6] Oxford, U.o . IncludED: A guide to designing inclusive assessments. 2024; Available from: https://www.ctl.ox.ac.uk/included-designing-inclusive-assessments#:~:text=Inclusive%20assessment%20design%20provides%20all,Tai%20et%20al%2C%202022

[7] Lucey CR , Hauer KE , Boatright D , Fernandez A . Medical education's wicked problem: achieving equity in assessment for medical learners. Acad Med. 2020;95(12S):S98‐S108. doi:10.1097/ACM.0000000000003717 32889943

[8] Tai J , Ajjawi R , Umarova A . How do students experience inclusive assessment? A critical review of contemporary literature. Int J Incl Educ. 2021;28(9):1‐18. doi:10.1080/13603116.2021.2011441

[9] de Oliveira E , Dantas RG , Amaral GA , Barreto Giaxa RR , de Góis AFT . Experiences of disabled students in undergraduate medical education. Med Teach. 2022;44(3):294‐299. doi:10.1080/0142159X.2021.1985098 34618650

[10] Woolf K , Rich A , Viney R , Needleman S , Griffin A . Perceived causes of differential attainment in UK postgraduate medical training: a national qualitative study. BMJ Open. 2016;6(11):e013429. doi:10.1136/bmjopen-2016-013429 PMC5168507 27888178

[11] Finn, G. , It's about more than end point assessment: a realist exploration of differential attainment in health professions education, in Ottawa. 2024: Melbourne, .

[12] Hauer KE , Park YS , Bullock JL , Tekian A . “My assessments are biased!” measurement and sociocultural approaches to achieve fairness in assessment in medical education. Acad Med. 2023;98(8S):S16‐S27. doi:10.1097/ACM.0000000000005245 37094278

[13] UCAS . Widening Access And Participation. 2024 [30 Jan 2024]; Available from: https://www.ucas.com/advisers/help-and-training/guides-resources-and-training/tools-and-resources-help-you/widening-access-and-participation

[14] Manchester, U.o . Addressing differential attainment. 2023 [14 February 2024]; Available from: https://www.bmh.manchester.ac.uk/about/equality/addressing-differential-attainment/

[15] Woolf K . Differential attainment in medical education and training. BMJ. 2020;368:m339. doi:10.1136/bmj.m339 32047006

[16] Office for Students . Our approach to equality of opportunity 2023; Available from: https://www.officeforstudents.org.uk/advice-and-guidance/promoting-equal-opportunities/our-approach-to-equality-of-opportunity/

[17] Saha S , Guiton G , Wimmers PF , Wilkerson L . Student body racial and ethnic composition and diversity‐related outcomes in US medical schools. JAMA. 2008;300(10):1135‐1145. doi:10.1001/jama.300.10.1135 18780842

[18] Harvey A , Burnheim C , Brett M . Towards a fairer chance for all: Revising the Australian student equity framework. In: Student Equity in Australian Higher Education: Twenty‐Five Years of a Fair Chance for All; 2016:3‐20.

[19] Commonwealth of Australia , Disability Discrimination Act. 1992.

[20] Commonwealth of Australia , Disability Standards for Education. 2005.

[21] Australian Human Rights Commission , A quick guide to Australian discrimination laws 2014.

[22] Education, M.o . Malaysia Education Blueprint 2013–2025. 2012.

[23] Soemantri D , Karunathilake I , Yang JH , et al. Admission policies and methods at crossroads: a review of medical school admission policies and methods in seven Asian countries. Korean J Med Educ. 2020;32(3):243‐256. doi:10.3946/kjme.2020.169 32723988 PMC7481048

[24] Samsudin EZ , Isahak M , Rampal S , Ismail R , Zakaria MI . Workplace bullying among junior doctors in Malaysia: a multicentre cross‐sectional study. Malays J Med Sci: MJMS. 2021;28(2):142‐156. doi:10.21315/mjms2021.28.2.13 PMC8075595 33958968

[25] Taras M . Assessment for learning: sectarian divisions of terminology and concepts. J Furth High Educ. 2008;32(4):389‐397. doi:10.1080/03098770802395892

[26] Norcini JJ , McKinley DW . Assessment methods in medical education. Teach Teach Educ. 2007;23(3):239‐250. doi:10.1016/j.tate.2006.12.021

[27] McLachlan JC , Robertson KA , Weller B , Sawdon M . An inexpensive retrospective standard setting method based on item facilities. BMC Med Educ. 2021;21(1):1‐7. doi:10.1186/s12909-020-02418-5 33407365 PMC7786895

[28] O'Shea S , Lysaght P , Roberts J , Harwood V . Shifting the blame in higher education–social inclusion and deficit discourses. High Educ Res Dev. 2016;35(2):322‐336. doi:10.1080/07294360.2015.1087388

[29] Sotiriadou P , Logan D , Daly A , Guest R . The role of authentic assessment to preserve academic integrity and promote skill development and employability. Stud High Educ. 2020;45(11):2132‐2148. doi:10.1080/03075079.2019.1582015

[30] Universities UK and n.u.o . Students, Black, Asian and minority ethnic student attainment at UK universities: #closingthegap. 2019.

[31] Times Higher Education , How universities can manage and monitor their edtech spend. 2022.

[32] Tai J , Mahoney P , Ajjawi R , et al. How are examinations inclusive for students with disabilities in higher education? A sociomaterial analysis. Assess Eval High Educ. 2023;48(3):390‐402. doi:10.1080/02602938.2022.2077910

[33] Tabish SA . Assessment methods in medical education. Int J Health Sci. 2008;2(2):3‐7. PMC3068728 21475483

[34] van der Vleuten CP , Schuwirth LW , Driessen EW , et al. A model for programmatic assessment fit for purpose. Med Teach. 2012;34(3):205‐214. doi:10.3109/0142159X.2012.652239 22364452

[35] Bearman M , Dawson P , Bennett S , et al. How university teachers design assessments: a cross‐disciplinary study. High Educ. 2017;74(1):49‐64. doi:10.1007/s10734-016-0027-7

[36] Battaglia A . Challenging the Essay Culture. King's Institute of Learning & Teaching; 2008:57.

[37] Tai JH‐M , Dollinger M , Ajjawi R , et al. Designing assessment for inclusion: an exploration of diverse students' assessment experiences. Assess Eval High Educ. 2023;48(3):403‐417. doi:10.1080/02602938.2022.2082373

[38] Firth M , Ball‐Smith J , Burgess T , Chaffer C , Finn G , Hansen J , Guy M , Havemann L , Glover N , Kingsbury M , Pazio M. Optionality in Assessment: a cross institutional exploration of the feasibility, practicality & utility of student choices in assessment in UK higher education. 2023.

[39] Jopp R , Cohen J . Choose your own assessment–assessment choice for students in online higher education. Teach High Educ. 2022;27(6):738‐755. doi:10.1080/13562517.2020.1742680

[40] The University Of Bristol vs. Dr Robert Abrahart, In Royal Courts Of Justice . 2024, The High Court Of Justice King's Bench Appeals Bristol District Registry.

[41] Morris, S. , Bristol University loses appeal over suicide of disabled student on exam day, in The Guardian 2024.

[42] Edmond MB , Deschenes JL , Eckler M , Wenzel RP . Racial bias in using USMLE step 1 scores to grant internal medicine residency interviews. Acad Med. 2001;76(12):1253‐1256. doi:10.1097/00001888-200112000-00021 11739053

[43] Stentiford L , Koutsouris G . What are inclusive pedagogies in higher education? A systematic scoping review. Stud High Educ. 2021;46(11):2245‐2261. doi:10.1080/03075079.2020.1716322

[44] Finn GM , Ballard W , Politis M , Brown ME . It's not alphabet soup–supporting the inclusion of inclusive queer curricula in medical education. Br Stud Dr J. 2021;5(2):27. doi:10.18573/bsdj.276

[45] McArthur J . Assessment for social justice: the role of assessment in achieving social justice. Assess Eval High Educ. 2016;41(7):967‐981. doi:10.1080/02602938.2015.1053429

[46] Muttaqin LH , Haekal M , Ibrahim I , Utami RT . Challenges and strategies for establishing an inclusive school in Indonesia: aligning Islamic values with inclusive education principles. Edukasi Islami: Jurnal Pendidikan Islam. 2023;12(3):2547‐2566.

[47] Finn, G. , Fostering inclusive curricula and learning environments: inclusivity reporting at a UK University Medical Science Educator, 2024. 10.1007/s40670-024-02049-1 PMC11296976 39099851

[48] Rahman AA , Woollard J . Neurodiversity awareness: is Malaysia there yet? Int J Eval Res Educ. 2019;8(4):676‐685.

[49] Zaidi Z , Rockich‐Winston N , Chow C , Martin PC , Onumah C , Wyatt T . Whiteness theory and the (in) visible hierarchy in medical education. Med Educ. 2023;57(10):903‐909. doi:10.1111/medu.15124 37199083

[50] Finn GM . Bygone Binaries: Considering Inclusion in Anatomy Education. Wiley Online Library; 2024. 10.1111/joa.14059 PMC11306757 38837754

[51] Finn, G. , Fostering inclusive curricula and learning environments: inclusivity reporting at a UK university Medical Science Educator, In press. 10.1007/s40670-024-02049-1 PMC11296976 39099851

